# *Houttuynia cordata* Thunb: An Ethnopharmacological Review

**DOI:** 10.3389/fphar.2021.714694

**Published:** 2021-09-01

**Authors:** Zhao Wu, Xinyu Deng, Qichao Hu, Xiaolin Xiao, Jing Jiang, Xiao Ma, Mingquan Wu

**Affiliations:** ^1^ State Key Laboratory of Southwestern Chinese Medicine Resources, Department of Pharmacy, School of Pharmacy, Chengdu University of Traditional Chinese Medicine, Chengdu, China; ^2^ Hospital of Chengdu University of Traditional Chinese Medicine, School of Clinical Medicine, Chengdu University of Traditional Chinese Medicine, Chengdu, China; ^3^ School of Physical Education, Chengdu University of Traditional Chinese Medicine, Chengdu, China; ^4^ Department of Pharmacy, Sichuan Orthopedic Hospital, Chengdu, China

**Keywords:** Houttuynia cordata thunb, anti-inflammatory, antiviral, immunomodulatory, antibacterial, antitumour

## Abstract

*Houttuynia cordata* Thunb (*H. cordata*; Saururaceae) is widely distributed in Asian regions. It plays an important role in traditional health care and disease treatment, as its aboveground stems and leaves have a long medicinal history in China and are used in the treatment of pneumonia and lung abscess. In clinical treatment, it can usually be combined with other drugs to treat dysentery, cold, fever, and mumps; additionally, *H. cordata* is an edible plant. This review summarizes detailed information on the phytochemistry and pharmacological effects of *H. cordata*. By searching the keywords “*H. cordata* and lung”, “*H. cordata* and heart”, “*H. cordata* and liver”, and “*H. cordata* and inflammation” in PubMed, Web of Science and ScienceDirect, we screened out articles with high correlation in the past ten years, sorted out the research contents, disease models and research methods of the articles, and provided a new perspective on the therapeutic effects of *H. cordata*. A variety of its chemical constituents are characteristic of medicinal plants, the chemical constituents were isolated from *H. cordata*, including volatile oils, alkaloids, flavonoids, and phenolic acids. Flavonoids and volatile oils are the main active components. In pharmacological studies, *H. cordata* showed organ protective activity, such as reducing the release of inflammatory factors to alleviate lung injury. Moreover, *H. cordata* regulates immunity, enhances the immune barriers of the vagina, oral cavity, and intestinal tract, and combined with the antibacterial and antiviral activity of its extract, effectively reduces pathogen infection. Furthermore, experiments *in vivo* and *in vitro* showed significant anti-inflammatory activity, and its chemical derivatives exert potential therapeutic activity against rheumatoid arthritis. Antitumour action is also an important pharmacological activity of *H. cordata*, and studies have shown that *H. cordata* has a notable effect on lung tumour, liver tumour, colon tumour, and breast tumour. This review categorizes the biological activities of *H. cordata* according to modern research papers, and provides insights into disease prevention and treatment of *H. cordata*.

## Introduction

Medicinal plants have a variety of chemical components and biological activities that can effectively prevent and treat common clinical diseases. In India and other Asian regions, people use natural plants to treat diseases, accounting for 70–95% of basic treatments (Drasar and Khripach, 2020). The health function of medicinal plants has also attracted attention, and flavonoids, saponins, polysaccharides and alkaloids isolated from plants show effective antiaging activities, which have great potential in the development of antiaging products (Shen et al., 2017). Investigating the active ingredients of plants that have medicinal use is also an ingenious way to develop drugs. The discovery of artemisinin, which was originally isolated from the plant *Artemisia annua*, is a good example (Fu et al., 2021).

**FIGURE 1:**
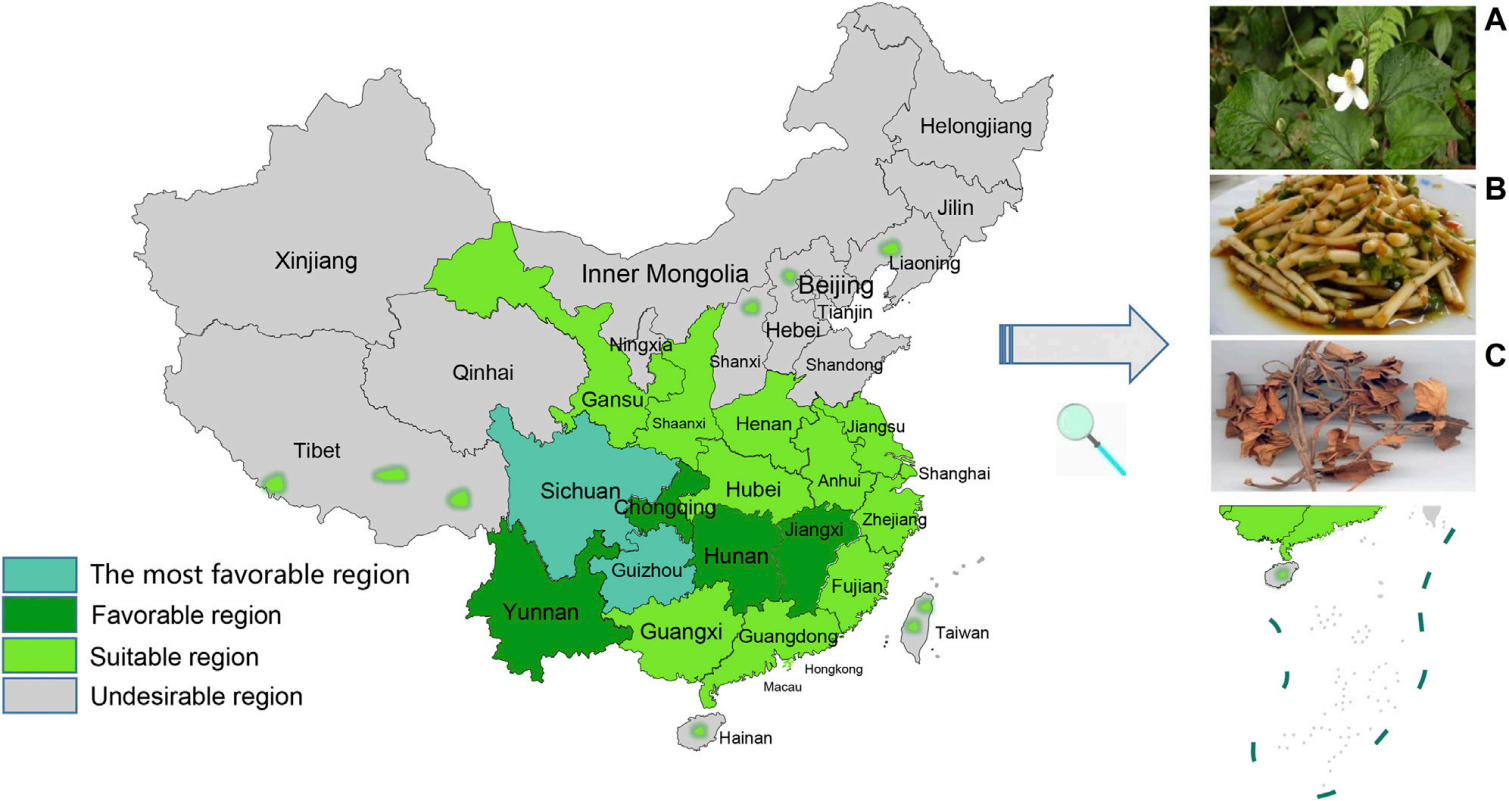
Climatic and ecological adaptability distribution of *H. cordata* in China **(A)** flowers of *H. cordata* **(B)** roots of *H. cordata* as food **(C)** above-ground part of dry stems and leaves of *H. cordata*.

*Houttuynia cordata* (*H. cordata*), a perennial herb, is a plant of the Saururaceae family that is widely used as a Chinese herbal medicine as well as a food. It prefers to grow in moist soil and warm environments. Its application has been described in China, Korea, Japan, India, and other Asian regions, and particularly in many provinces in China (Figure 1). *H. cordata* has been eaten and used as medicine by local people for the past few thousand years. Currently, it is also harvested for daily food and medicine in the Yarlung Zangbo Valley in Assam, India (Kumar et al., 2014).

In general, the whole plant of *H. cordata* can be used as medicine, and it is applied by considering traditional Chinese medical theory, allowing it to be used to treat pneumonia caused by viral infection in combination with Forsythia and Magnolia (Park et al., 2005). Moreover, effective antiviral activity has been reported in many studies, and *H. cordata* has a significant inhibitory effect on virus infection and replication (Muluye et al., 2014). In the severe acute respiratory syndrome (SARS) virus infection outbreak in 2003, *H. cordata* was listed as one of the drugs for the treatment of SARS (Lau et al., 2008).

The disease prevention and potential treatment of *H. cordata* cannot be realized without effective research. Therefore, the objective of this review is to provide a unified governance framework that guides studies on the pharmacological activity of *H. cordata* and its derivatives *in vivo* and *in vitro*. This is achieved by reviewing the therapeutic activity of *H. cordata* in different organs, tissues and cells in the past research. This study is also an attempt to synthesize scattered sources of information obtained regarding the therapeutic activity of *H. cordata* against diseases.

## Chemical Composition

*H. cordata* contains a variety of chemical components (Table 1), and alkaloids were the most abundant ingredients (Ahn et al., 2017); however, volatile oil and flavonoids were the main components that exerted pharmacological activity. Interestingly, the decanoyl acetaldehyde component has a special fishy scent, so it is called Yu-Xing-Cao (traditional Chinese medicinal herb) in China (Ma et al., 2017), and it is also an antibacterial active ingredient and is easily converted to 2-undecanone (methyl n-nonanone) at high temperature, which can be used to evaluate the quality of *H. cordata* oil to a certain extent (Chen et al., 2014). Moreover, its steam distillation extract contained essential oils including monoterpenes, sesquiterpenes and their oxides, oxidized diterpenes and phenylpropene derivatives (Řebíčková et al., 2020); and nonyl ketones (2.10–40.36%), bornyl acetate (0.4–8.61%) and β-myrcene (2.58–18.47%) are the main components in essential oils (Lu et al., 2006). Interestingly, there were differences in the contents of the aboveground stems and the underground parts, as the contents of 2-undecanone, myrcene, ethyl decanoate, ethyl dodecanoate, 2-tridecanone and decanal in the aboveground parts were higher than those in the underground parts; In particular, 11 ingredients were only isolated in the leaves, while seven ingredients in the roots were not contained in the leaves. Interestingly, there seemed to be variation in different regions. Researchers also reported differences in the antibacterial activity of *H. cordata* from different areas, but these findings still lack sufficient support (Verma et al., 2017).

**TABLE 1:** Important chemical compositions of *Houttuynia cordata*.

Species	Serial number	Ingredients	Molecular formula	Molecular weight	References
Volatile oils	(a)	Houttuynin	C12H22O2	198.30	Chen et al. (2014)
	(b)	Bornyl acetate	C12H20O2	196.29	Lu et al. (2006)
	(c)	β-Myrcene	C10H16	136.23	Lu et al. (2006)
	(d)	Ethyl caprate	C12H24O2	200.32	Verma et al. (2017)
	(e)	Ethyl dodecanoate	C14H28O2	228.38	Verma et al. (2017)
Flavonoids	(f)	2-Undecanone	C11H22O	170.30	Verma et al. (2017)
	(g)	2-Tridecanone	C13H26O	198.35	Verma et al. (2017)
	(h)	Rutinum	C27H30O16	610.52	Xu et al. (2006)
	(i)	Hyperoside	C21H20O12	464.38	Xu et al. (2006)
	(j)	Quercitrin	C21H20O11	448.38	Lu et al. (2011)
	(k)	Quercetin	C15H10O7	302.24	WU et al. (2009)
	(l)	Houttyunoid A	C33H38O13	665.22	Chen et al. (2012); Chen S. D. et al. (2013)
	(m)	Houttyunoid B	C32H38O12	637.23	Chen et al. (2012); Chen S. D. et al. (2013)
	(n)	Houttyunoid C	C33H38O13	665.22	Chen et al. (2012); Chen S. D. et al. (2013)
	(o)	Houttyunoid D	C32H40O13	655.24	Chen et al. (2012); Chen S. D. et al. (2013)
	(p)	Houttyunoid E	C32H40O13	655.24	Chen et al. (2012); Chen S. D. et al. (2013)
Phenolic acids	(q)	Linolenic acid	C18H32O2	1,280.44	Bauer et al. (1996)
	(r)	Linoleic acid	C18H32O2	280.44	Bauer et al. (1996)
	(s)	Oleic acid	C18H34O2	282.46	Bauer et al. (1996)
	(t)	Palmitic acid	C16H32O2	256.42	Bauer et al. (1996)
	(u)	Stearic acid	C18H36O2	284.48	Bauer et al. (1996)
	(v)	Neochlorogenic acid	C16H18O9	354.31	Nuengchamnong et al. (2009)
	(w)	Chlorogenic acid	C16H18O9	354.31	Nuengchamnong et al. (2009)
	(x)	4-Dicaffeoylquinic Acid	C16H18O9	354.31	Nuengchamnong et al. (2009)
Alkaloids	(y)	Aristololactam	C17H11NO4	293.27	Ma et al. (2017)
	(z)	Houttuynoside A	C21H22O11	450.40	Chou et al. (2009)
	(aa)	Houttuynamide A	C15H15NO2	273.10	Chou et al. (2009)

Moreover, the flavonoids in *H. cordata* include rutin, hyperoside, quercetin, and quercitrin, and most of them are combined with rhamnose in the form of glycosides (Xu et al., 2006; WU et al., 2009; Lu et al., 2011). Chen et al. isolated a new combination of houttuynin and hyperoside (houttuynoids A-E [1–5]), four new flavonoid compounds (Chen et al., 2012; Chen S. D. et al., 2013), and Chou et al. isolated the houttuynoside A and houttuynamide A (Chou et al., 2009). However, phenolic acids are the most isolated components in *H. cordata*, including linolenic acid, linoleic acid, oleic acid, palmitic acid, stearic acid (Bauer et al., 1996), quinic acid derivatives, caffeic acid derivatives, neochlorogenic acid, chlorogenic acid, cryptochlorogenic acid and other ingredients (Nuengchamnong et al., 2009).

**FIGURE 2:**
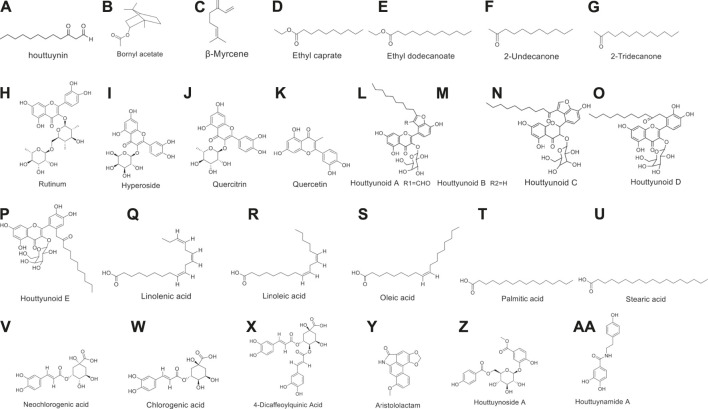
Chemical structures of important constituents of *H. cordata*.

Furthermore, alkaloids are the key components of the physical activity of houttuynia-containing herbs, most of which are phenanthrolactam compounds such as aristololactam and piperolactam (Probstle and Bauer, 1992; Ma et al., 2017). The structural formulas of above compounds are shown in Figure 2.

## Pharmacological Activity

### Lung Protection

Lung diseases have various causes. General symptoms are inflammation of the lungs accompanied by acute lung injury (ALI). The anti-inflammatory activity of *H. cordata* plays an indispensable role in alleviating lung diseases, which might be associated with its flavonoids, sodium houttuyfonate and polysaccharides, in a lipopolysaccharide (LPS)-induced model, proinflammatory cytokine (IL-6) and NO production were obviously reduced by oral administration of quercitrin from *H. cordata* at 100 mg/ml (Lee et al., 2015)*.* Interestingly, in a model of chronic obstructive pulmonary disease (COPD) induced by LPS combined with cigarette smoke for 4 weeks, 24.3 mg/kg sodium houttuyfonate decreased the mRNA levels of TLR4, MyD88, and NF-κB p65 (Wu et al., 2017), however, considering the differences in the adaptability of humans and rats to cigarette smoke, this research still needs more investigation. Regardless, macromolecular polysaccharides contribute to alleviating lung injury by reducing pulmonary oedema and protein exudation of bronchoalveolar lavage fluid (Lu et al., 2018). Cell transplantation combined with *H. cordata* has also been used to treat lung tissue injury. Injection of 50 mg/g extract via the tail vein downregulated the inflammatory response and reduced the expression of iNOS and ET-1, thereby enhancing the therapeutic effect of endothelial progenitor cells on LPS-induced ALI in rats (Cai et al., 2013). Furthermore, in an acute lung tissue injury model caused by H1N1 virus infection, utilizing 50, 100, and 200 mg/kg *H. cordata* flavonoid glycoside extract compared to 100 mg/kg ribavirin resulted in less weight loss and a lower lung index in 14 days. Antibacterial and anti-inflammatory activity were realized by inhibiting H1N1 neuraminidase activity and the expression of toll-like receptors (TLRs) (Ling et al., 2020), and regulation of polysaccharide on gut mucosal-associated lymphoid tissue (GALT) might be the mechanism that alleviates ALI caused by influenza A virus, as it downregulates Th17 cell differentiation and upregulates Treg cell differentiation to restore Th17/Treg balance from the GALT to the lung, reducing IL-17A and increasing IL-10 to alleviate lung mucosal damage (Shi et al., 2020).

Previous studies have confirmed that pulmonary fibrosis is associated with lung oxidative damage. In bleomycin-induced pulmonary fibrosis in rats, a water extract of *H. cordata* significantly reduced the concentrations of superoxide dismutase, malondialdehyde, and hydroxyproline, showing stronger antioxidant activity than vitamin E. Otherwise, *H. cordata* can relieve the pathological changes of lung tissue caused by bleomycin (Ng et al., 2007), and the increasing level of IFN-γ and inhibition of the TGF-β1/Smad signalling pathway might be a significant mechanism. Meanwhile, 4-terpineol, α-terpineol, L-bornyl acetate and methyl-n-nonyl ketone were significantly decreased in a dose-dependent manner at doses of 3.5–16.5 mg/kg *in vivo*. *In vitro*, the expression of TGF-β1 was inhibited in a dose-dependent manner, and IFN-γ levels were also upregulated in NIH/3T3, thus alleviating the lung fibrosis induced by LPS (Du et al., 2012).

Overall, the above studies revealed that the protective effect of *H. cordata* is related to its anti-inflammatory active ingredients, which mainly include flavonoids, polysaccharides, and sodium houttuyfonate, although the dominant component has not been reported. Mice show consistency in certain symptoms of respiratory diseases with humans, but there are significant differences in the immune system (Shilovskiy et al., 2021), so it is difficult to judge whether the above research results act through the same mechanism in humans.

### Digestive System Protection

#### Reduction of Intestinal Injury

There are mucosal barriers in the intestine to avoid damage, which generally include mechanical barriers, chemical barriers, immune barriers, and biological barriers. In addition, the intestinal flora plays an important role in protecting the intestines (Lu et al., 2019). Recent studies have found that polysaccharides and sodium houttuyfonate in *H. cordata* protect the intestinal flora, a crude polysaccharide extract significantly reduces intestinal goblet cells, and the expression of sIgA and tight junction protein (ZO-1) in the intestine is upregulated to strengthen the intestinal mechanical barriers and immune barrier (Zhu et al., 2018). In a mouse model of intestinal inflammation caused by *Salmonella typhimurium*, Zhang et al. demonstrated the protection of sodium houttuyfonate in restoring the intestinal barrier by regulating the tissue distribution of tight junction proteins, and inflammation was also reduced by inhibiting the NF-κB signalling pathway (Zhang et al., 2020). However, such barrier-enhancing activity lacks effective control. Moreover, the regulation of bacteria is also involved, and polysaccharides composed of Glc, Gal, Ara, and Rha at a 40 mg/kg dosage greatly reduced the relative abundance of the pathogenic bacteria *Vibrio* and *Bacillus* and alleviated the intestinal damage caused by H1N1 infection (CHEN et al., 2019). These findings indicated that *H. cordata* polysaccharides and sodium houttuyfonate exert intestinal protective activity by regulating the intestinal flora and inhibiting the NF-κB signalling pathway to strengthen the intestinal barrier.

#### Reduction of Liver Damage

Recently, natural products in plants have shown effective activity in the prevention and treatment of liver diseases. Researchers have confirmed that natural products such as terpenoids, alkaloids, glycosides, and coumarins inhibit liver fibrosis (Ma et al., 2020a); moreover, rutin and quercetin show potential therapeutic activity in cholestasis (Ma et al., 2020b). Liver cells are sensitive to oxidative stress, and the ethyl acetate extract of *H. cordata*, which confers liver protection, showed significant antioxidant activity in a CCl_4_-induced liver injury mice model. In particular, the 1,000 mg/kg extract remarkably inhibited the increase of glutathione, superoxide dismutase and catalase; furthermore, the levels of serum transaminase and liver malondialdehyde also decreased in mice (Tian et al., 2012), and in ethanol-induced liver damage model, the CYP2E1 activity of mice treated with the mixture of *H. cordata* water and ethanol extract at 300 mg/kg/day for 7 days was significantly reduced, thereby decreasing the level of oxidative factors that CYP2E1 mediated, while the expression of antioxidant enzymes and lipogenic mRNA was increased (You et al., 2018). Overall, in the liver oxidative damage model, *H. cordata* inhibited the increase of glutathione, superoxide dismutase and catalase and regulated the release of oxidative factors mediated by CYP2E1 to relieve oxidative damage. However, the dosages used in different models are not uniform, and whether high-dose *H. cordata* extract has a toxicity needs to be investigate.

### Heart Protection

Antioxidant components of *H. cordata* show interventional activity in the process of heart remodelling and functional decline. In diabetic mice, continuous intake of 2% *H. cordata* water extract for 8 weeks downregulated cardiac active oxygen, protein carbonyl, interleukin-6 and inflammatory factors; moreover, intake of 1 and 2% *H. cordata* water extract inhibited the expression of p47^phox^, NF-κB p65 and p-p38 in the heart caused by diabetes (Hsu et al., 2016). With further research, similar activity of sodium houttuyfonate was found in an isoprenaline-induced myocardial hypertrophy model treated with 90 and 180 mg/kg sodium houttuyfonate for 1 week, and the results showed that the concentration of cyclic adenosine monophosphate, heart weight index, left ventricular weight index, and angiotensin II were simultaneously reduced. In the l-thyroxine-induced cardiac hypertrophy model, the expression of hydroxyproline and the cross-sectional area of cardiomyocytes were downregulated. This myocardial protection might be associated with the suppression of the sympathetic nervous system, renin-angiotensin system and endothelin expression (Gao et al., 2009). Additionally, the activation of the renin-angiotensin-aldosterone system was attenuated by sodium houttuyfonate at dosages of 50 and 100 mg/kg, with the activity of alleviating cardiac inflammation and fibrosis; and it showed inhibition of ventricular remodelling in a model of abdominal artery stenosis-induced ventricular remodelling in rats (Gao et al., 2010). Moreover, the anti-ventricular remodelling mechanism after myocardial infarction of sodium houttuyfonate might be associated with the adenosine monophosphate-activated protein kinase (AMPK) activation and NF-κB pathway inhibition at the same dosage; moreover, the release of myocardial inflammatory factors also declines, thereby relieving the fibrosis process (Zheng et al., 2018). However, in the anti-angiogenic research of *H. cordata*, no effective activity was seen in a zebrafish model (Tu et al., 2016).

Taken together, the *H. cordata* extract reduced the release of inflammatory factors to alleviate myocardial oxidative damage. The sodium houttuyfonate component can affect the sympathetic nervous system and the renin-angiotensin-aldosterone system to reverse myocardial hypertrophy and remodelling, and the molecular mechanism may relate to AMPK activation inhibited.

### Kidney Protection

Kidney diseases are usually caused by inflammation, oxidative damage and other factors that seriously affect the body’s water and salt metabolism (Gong et al., 2020). In diabetic mice with kidney injury, treatment with 1 and 2% *H. cordata* water extract reduced the level of urea nitrogen and the activity of creatine kinase, and the expression of kidney oxidative factors also decreased. Meanwhile, intake of 2% extract seemed to restrict the expression of membrane-anchored receptor of advanced glycation end products (RAGE), which can induce intracellular reactive oxygen species generation and activate mitogen-activated protein kinase (MAPK) and NF-κB signalling pathways, indicating its renal protective activity (Hsu et al., 2016). Inflammation is also an important mechanism leading to kidney damage. Research by Pan et al. showed that the sodium houttuyfonate significantly reduced the expression of nuclear NF-κB and MCP-1 in a dose-dependent manner at 60–120 mg/kg. In turn, it inhibited cationic bovine serum albumin-induced membranous glomerulonephritis and exhibited renal protective activity (Pan et al., 2010). In summary, the findings suggested that *H. cordata* extract and sodium houttuyfonate reduced kidney oxidative stress damage and inflammation through the glycation polyol pathway, downregulating the expression of NF-κB and MCP-1 and thereby alleviating kidney damage.

### Antitumour Activity

#### Anti-Lung Tumour Activity

Lung cancer usually includes small cell lung cancer (SCLC) and non-small-cell lung cancer (NSCLC). Generally, inducing tumour cell apoptosis and inhibiting tumour cell migration play a vital role in the treatment of lung tumour (Jones et al., 2018). Researchers demonstrated the antitumour activity of *H. cordata* and its active ingredient 2-undecanone in a study of benzo(a)pyrene-induced lung tumour in mice, and activation of the Nrf2-HO-1/NQO-1 pathway might be involved, which in turn inhibits lung cell DNA damage and inflammation. Moreover, there was no obvious systemic toxicity in mice (Lou et al., 2019). In addition, the polysaccharides in *H. cordata* have been found to possess antitumour activity, and the isolated pectin polysaccharide HCA4S1 has been experimentally proven to inhibit tumour cell proliferation by inducing A549 lung tumour/cancer cell cycle arrest and apoptosis. At the same time, the activities of cleaved caspase3 and cyclin B1 in cells after HCA4S1 treatment were significantly increased (Han et al., 2018). Chen et al. further investigated the apoptosis mechanism of lung tumour cells, and the active ingredients of *H. cordata* blocked cell proliferation by acting on the G0/G1 phase of A549 cells. In addition, Fas/CD95 protein levels in A549 cells were upregulated, and caspase-8 and caspase-3 were activated (Chen Y. F. et al., 2013). In another study on the migration of NSCLC, sodium houttuyfonate in *H. cordata* was found to inhibit the migration of tumour cells, and the inhibition of Linc00668 activity might be a vital feature, resulting in the decrease in Slug mRNA levels regulated by miR-147a. This revealed the important role of the Linc00668/miR-147a/slug axis in inhibiting lung tumour cell migration (Jiang et al., 2019). The above research results indicated that *H. cordata* might alleviate DNA damage by activating the Nrf2-HO-1/NQO-1 pathway, blocked cell proliferation by acting on the G0/G1 phase, and regulated the level of lncRNAs to inhibit tumour cell migration. However, the above findings were mainly investigated *in vitro,* and more *in vivo* investigations are still needed.

#### Anti-Liver Tumour Activity

In a study of human HepG2 cells exposure to high glucose, *H. cordata* extract at a concentration range of 0–80 μg/ml reduced lipid accumulation in HepG2 cells in a dose-dependent manner. This mechanism is related to inhibition of the AMPK signalling pathway, reduction of AMPK-mediated lipid synthesis, and alleviation of the proliferation of liver tumour cells (Kang and Koppula, 2014). Moreover, the apoptosis-inducing activity of *H. cordata* has been investigated, and the levels of factor (HIF)-1A, Forkhead box (FOX)O3, and MEF2A were significantly upregulated in human HepG2 hepatocellular carcinoma cells. At the same time, *H. cordata* enhanced the expression of caspase-3 and caspase-7 through MEF2A, while Bax, Bcl-2 and Bcl-xL protein levels were also disturbed, thus inducing apoptosis of liver tumour cells (Kim et al., 2017). Taken together, lipid accumulation in human HepG2 cells can be reduced after treatment with *H. cordata*, and HIF-1A, FOXO3, and MEF2A factors are significantly activated; however, the chemicals that act to induce apoptosis in liver tumour and investigation in animal models are still needed.

#### Anti-Colon Tumour Activity

In research on products for the treatment of colon tumour, Tang et al. found that an ethanol extract of *H. cordata* showed antitumour activity against the colon tumour cell line HT-29. Treatment with a 450 μg/ml extract can significantly induce apoptosis of tumour cells, increase reactive oxygen species and decrease the mitochondrial membrane potential. In particular, cytochrome c, Apaf-1, pro-caspase-9 and AIF are released from mitochondria due to changes in membrane potential by Western blotting and caspase activity assays. This result revealed the mitochondria-dependent mechanism by which *H. cordata* extract induces apoptosis of HT-29 cells (Tang, et al., 2009). Moreover, the investigation of induced cytotoxicity in primary colorectal cancer/tumour cells showed the same results; mitochondrial-dependent apoptosis mechanisms were also involved, and the production of reactive oxygen species increased. After treatment with 250 μg/ml *H. cordata* extract for 24 h, primary colorectal cancer/tumour cells showed chromosome condensation and apoptosis (Lai et al., 2010). Taken together, the molecular mechanism of the cytotoxicity of *H. cordata* extract mainly consists of reducing the mitochondrial membrane potential, thereby increasing the levels of cytochrome c, Apaf-1, caspase-3 and -9 and inducing cancer/tumour cell apoptosis, and the cytotoxicity to colon cells is still lacking.

#### Anti-Gastric Carcinoma Activity

Gastric carcinoma is the third most fatal tumour and is a prevalent malignancy worldwide, with approximately 1,033,701 new cases reported annually (Bray et al., 2018). The induce apoptosis and inhibit migration activity of herbs at the interface of food and medicine was investigated in gastric carcinoma, and a food composed of six plants, *Coix seed, Lentinula edodes*, *Asparagus officinalis* L., *H. cordata*, *Taraxacum mongolicum* Hand.-Mazz., and *Grifola frondose*, was used to treat gastric carcinoma in nude mice inoculated with SGC-7901 cells; supplementing 43.22, 86.44, and 172.88 g/kg food for 30 days, the serum levels of MMP-2 and MMP-9 decreased, while TNF-α significantly increased. Moreover, treatment notably upregulated the mRNA expression levels of GSK-3β, E-cadherin, Bax, Caspase-3, and Caspase-9 and the Bax/Bcl-2 ratio but substantially downregulated β-catenin, N-cadherin, MMP-2, MMP-9, Snail, and Cyclin D1, especially Ki-67 and N-cadherin, in tumour tissues. The underlying molecular mechanism might be associated with inhibition of the Wnt/β-catenin signalling pathway (Chen X. et al., 2021); this finding indicated the possibility of *H. cordata* forming synergistic interactions with the other five plants to prevent gastric carcinoma as a daily food; however, the specific activity of *H. cordata* among it has not been investigated. Interestingly, researchers compared the anti-gastric carcinoma activity of heated and unheated parts of *H. cordata* separately through DAPI staining and the detection of apoptosis and apoptotic protein levels, and cell viability decreased in SGC-7901, HepG2, NCI-H640, and HO-8910 cells with the increase of the extract concentration at 0, 25, 50, 100, and 150 ml/L. Furthermore, heated ariel stems showed 3–15 times higher effects than stems that were not heated in SGC-7901 cells, and the morphological characteristics of apoptosis, p53 protein, pro-apoptotic protein Bax, Bid, Bak, Apaf-1, activation of PARP, caspase9, and caspase3 were also increased, and the heated sample seemed to have higher activity than the unheated sample (Liu et al., 2020). The above findings suggested the potential of *H. cordata* in preventing gastric carcinoma in the diet.

#### Other Anti-Tumour Activity

The overexpression of HER2/neu has been shown to be related to breast cell canceration (Dawood et al., 2010; de la Cruz-Merino et al., 2017), which suggests that it may be an anti-breast tumour target. In the research by Zhou et al., houttuyfonate, the active ingredient of *H. cordata*, was modified by adding sulfhydryl groups and showed a significant reduction in tumour volume. At a concentration of 5.52 μg/ml, HER2 phosphorylation and HER2/neu-mediated activation of ERK1/2 and AKT were inhibited (Zhou et al., 2012). In addition, studies have shown that the ethanol extract of *H. cordata* induced breast tumour cell apoptosis. At concentrations of 100–500 μg/ml, MCF-7 and MDA-MB-231 cells stagnated in the G1 phase, and this result might be related to the downregulation of cyclin D1 and CDK4 expression at low concentrations. Moreover, the secretion of MMP-2 and MMP-9 is significantly inhibited, thereby inhibiting the migration and invasion of tumour cells (Subhawa et al., 2020). Overall, *H. cordata* and its derivatives seem to exert anti-breast tumour activity by suppressing tumour volume, inducing apoptosis and inhibiting migration. The regulation of HER2/neu overexpression and cell cycle arrest was also involved.

**FIGURE 3:**
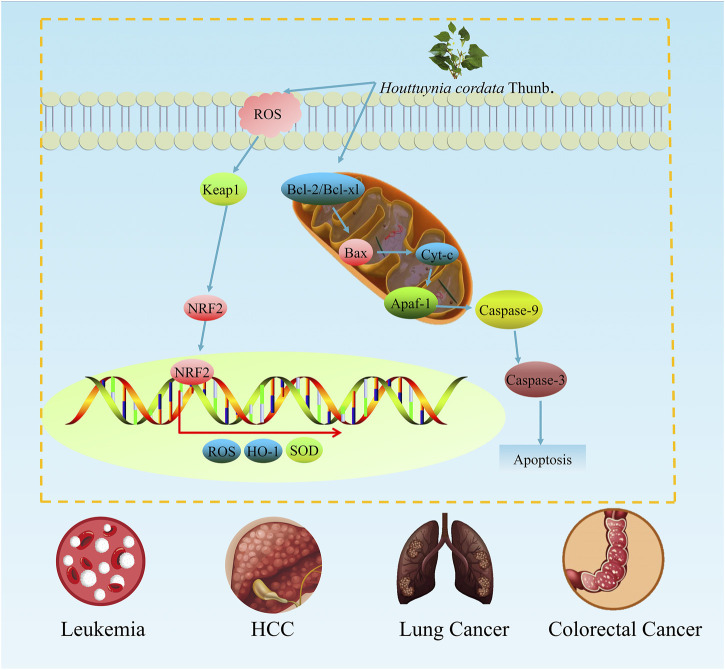
The antitumour effects of *H. cordata. H. cordata* might suppress multiple tumours mainly by inhibiting the NRF2 signalling pathway and promoting the process of apoptosis.

Leukaemia is a type of disease caused by the malignant cloning of haematopoietic stem cells. In early studies, the ingredients of *H. cordata*, caffeic acid has been found to induce apoptosis in leukaemia cells. At a concentration of 45 μM, caffeic acid treatment for 2 days significantly reduced the activity of U937 leukaemia cells. Additionally, as a typical apoptotic feature, the cleavage of PARP and procaspase-3 was obviously activated, and the apoptotic rate of leukaemia cells treated with 100 mM caffeic acid reached 59.87% (Jang et al., 2011). Furthermore, a study in leukaemic Moult-4 cells revealed the molecular mechanism by which *H. cordata* extract induces apoptosis, the alcohol extract of *H. cordata* decreased the mitochondrial transmembrane potential, the expression of Bcl-xl was downregulated, and the protein levels of Smac/Diablo, Bax and GRP78 increased (Prommaban et al., 2012). In conclusion, *H. cordata* can increase the lysis of PARP and procaspase-3 in leukaemia cells and induce Moult-4 cell apoptosis through the endoplasmic reticulum stress pathway (Figure 3).

### Anti-Inflammatory Effects and Immunomodulatory Activity

#### Anti-Inflammatory Effects

The occurrence of inflammation is related to a variety of cells, such as eosinophils, basophils, neutrophils, macrophages, monocytes, and mast cells (Roe, 2021). Mast cells play a vital role in mediating inflammatory diseases such as asthma and allergies. Interestingly, the ability of an extract of *H. cordata* to inhibit mast cell-mediated inflammatory diseases was investigated by Kim’s et al.; the mast cell line HMC-1 was treated with the ethyl acetate *H. cordata* extract at a concentration of 10 μg/ml, and the chemotactic index, secretion, and mRNA levels of inflammatory factors TNF-α and IL-6 were downregulated. In addition, stem cell factor-mediated NF-κB activation was inhibited (Kim et al., 2007; Lee et al., 2013). Moreover, in interstitial bladder inflammation, not only are proinflammatory factors decreased, but the proliferation and activation of mast cells are effectively attenuated, demonstrating the potential value of *H. cordata* in the treatment of interstitial cystitis (Li et al., 2020).

In a RAW264.7 cell experiment induced by LPS, both 2-methyl nonyl ketone and sodium houttuyfonate components isolated from *H. cordata* showed anti-inflammatory activity, and expression of tumour necrosis factor-α (TNF-α), interleukin-1β (IL-1β) and Toll-like receptor 4 (TLR4) were decreased while the level of interleukin-10 (IL-10) was upregulated. Moreover, the supercritical extract of *H. cordata* inhibited RAW 264.7 cell inflammation through the TNF-α-NO and cyclooxygenase II-PGE_2_ pathways, and the extract at a dose of 200 mg/kg significantly reduced inflammatory cells and albumin exudation after oral administration (Shin et al., 2010). In addition, its fermentation products showed similar activity; the expression of the proinflammatory factors PGE_2,_ iNOS, IL-1β, TNF-α and IL-6 was downregulated, while the effect on COX-2 activity was weak (Woranam et al., 2020). Furthermore, in LPS-induced peritoneal macrophages, the supercritical extract of *H. cordata* showed similar effects to nonsteroidal anti-inflammatory drugs (NSAIDs) and the COX-2 inhibitor NS398; it not only reduced COX-2 enzyme activity in a dose-dependent manner but also downregulated COX-2 mRNA and protein expression (Li et al., 2011). At the same time, tumour necrosis factor-α (TNF-α) mRNA expression and NO factor levels induced by LPS were significantly inhibited by *H. cordata*. *In vivo*, xylene-induced ear swelling and inflammation in mice were significantly inhibited, and sodium houttuyfonate showed stronger anti-inflammatory activity than 2-methyl nonyl ketone (Chen et al., 2014). Moreover, mouse foot swelling induced by formaldehyde and carrageenan was also effectively relieved by the volatile oil components of *H. cordata* (Li et al., 2013).

**FIGURE 4:**
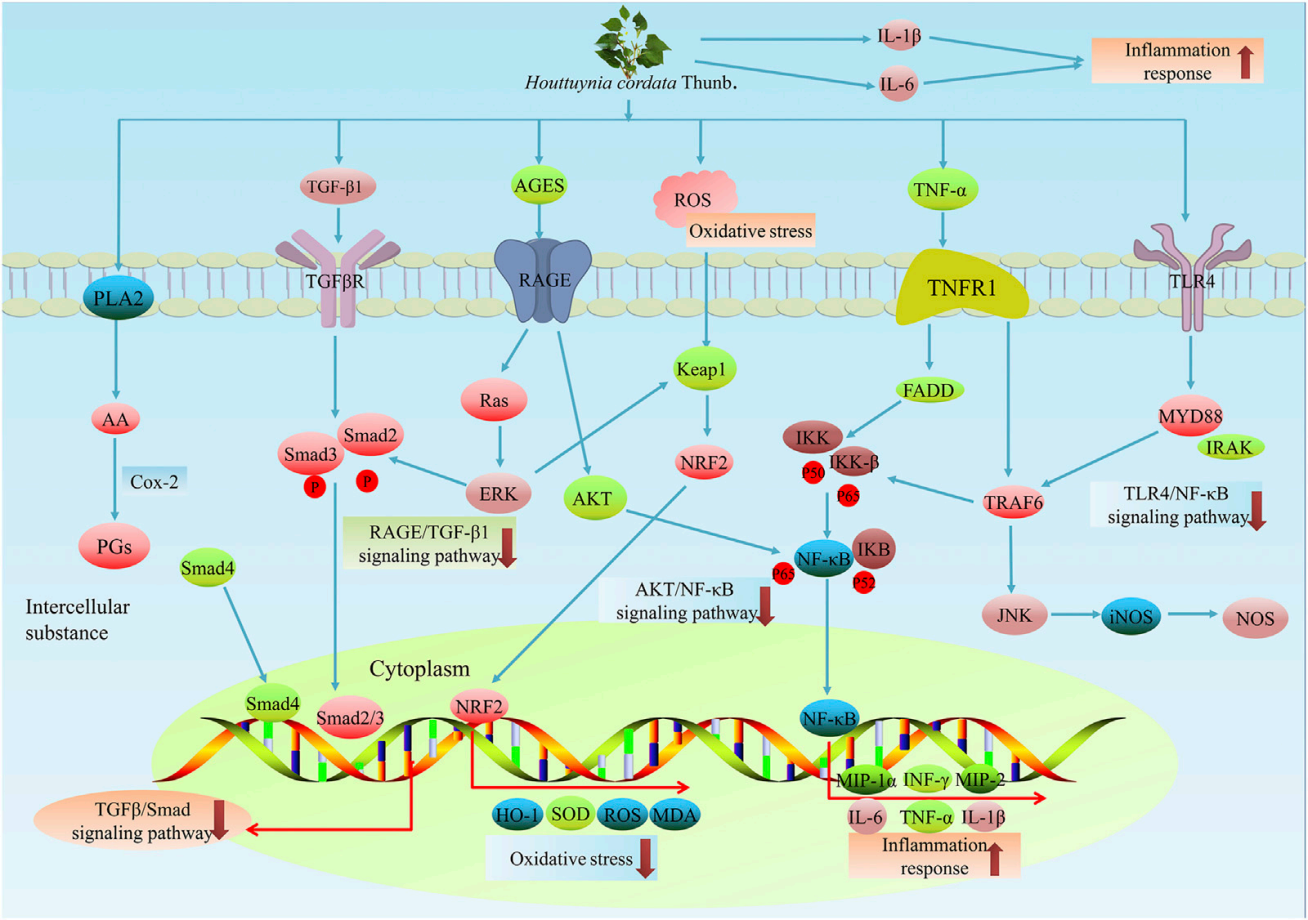
The anti-inflammatory effects of *H. cordata*. *H. cordata* suppresses inflammation mainly via several signalling pathways. H. cordata reduces the production of PGs by acting on PLA2. In addition, *H. cordata* seemed to suppress inflammation by downregulating RAGE/TGFβ1 signals, AKT/NF-κB signals, TLR4/NF-κB signals and TGF-β/Sma d signals.

Interestingly, inflammation of human keratinocytes was also alleviated by the *H. cordata* ethanol extract, and the secretion of interleukin-8, CCL20, IP-10, and GRO-α caused by *Porphyromonas gingivalis* was effectively reduced after treatment (Figure 4). These research results suggested the application of *H. cordata* in oral infections (Sekita et al., 2017). Sodium houttuyfonate also showed potential therapeutic activity for rheumatoid arthritis, which is the destruction of the joint caused by pathological hyperplasia of the synovium. Through *in vitro* synovial cell experiments, sodium houttuyfonate effectively inhibited the proliferation of synovial cells in a dose-dependent manner within the range of 25 μg/ml∼200 μg/ml (Li et al., 2014; Li and Zhao, 2015).

#### Immunomodulatory Activity

Immune cells, including neutrophils, eosinophils, basophils, and mast cells, play a vital role in ensuring immune function. Immune active substances such as immunoglobulin, interferon, tumour necrosis factor, and interleukin also play important roles (Sattler, 2017). Allergy is a common immune function abnormality that can cause serious diseases such as anaphylactic shock, microcirculation disorders, and central nervous system disorders (Marshall, 2018).

The polyphenols in *H. cordata* have shown antiallergic activity. After basophilic KU812F cells were cultured with extract, the FcεRI level and IgE binding activity decreased significantly. In addition, the mRNA activity of FcεRI-α and γ-chains was also downregulated, and the release of histamine was restricted (Shim et al., 2009). *In vivo*, oral *H. cordata* extract can effectively alleviate passive cutaneous anaphylaxis in mice. FcεRI signalling molecules activated by antigens such as Syk, Lyn, LAT, Gab2, and PLCγ2 and downstream Akt and MAP kinases were also inhibited, but the level of cAMP in mast cells increased, which reveals that *H. cordata* can improve allergic diseases by inhibiting the FcεRI-dependent signal transmission of mast cells (Li et al., 2005; Han et al., 2009). HCP-2, a polysaccharide isolated from *H. cordata*, regulated the expression of T cells in human peripheral blood mononuclear cells at concentrations of 0.1–25 μg/ml, and the levels of the immune molecules interleukin-1β (IL-1β), tumour necrosis factor-α (TNF-α), and macrophage inhibitory protein-1α and -1β increased significantly; accordingly, the body’s immunity was effectively enhanced (Cheng et al., 2014). Moreover, researchers determined the therapeutic effect of *H. cordata* extract on Th2-mediated immune diseases. Ethanol extract not only inhibited the production of TARC in skin fibroblast CCD-986sk cells but also downregulated the level of TARC receptor CC chemokine receptor 4 (CCR4) mRNA in Jurkat T cells, and the migration of T cells induced by TARC was also restricted (Lee et al., 2008). In addition, *H. cordata* extract regulates innate immune mediators. After vaginal epithelial cells were treated with the extract for 18 h, the mRNA levels of human β-defensin 2 and secretory leukocyte protease inhibitor increased remarkably, IL-2 and IL-6 protein secretion increased, and CCL5 secretion decreased (Satthakarn et al., 2015b). Interestingly, the extract has similar effects on oral immune mediators, and the expression of human β-defensin 2, secretory leukocyte protease inhibitor, IL-8 and CCL20 is regulated by the extract in a dose-dependent manner (Satthakarn et al., 2015a). The above results showed that vaginal and oral immune mediators are upregulated by *H. cordata* extract, suggesting the potential of *H. cordata* to prevent and treat oral diseases in diet.

### Antiviral Activity

#### Anti-Herpes Virus Activity

*H. cordata* displays obvious activity in inhibiting herpesvirus, its solution extracted with hot water effectively attenuated herpes simplex virus (HSV) infection, which might be associated with the inhibition of the NF-κB pathway; however, the activity of another key pathway, Erk MAPK, was not regulated. Moreover, determination of the anti-infective activity of the powder after lyophilization of the extract revealed the IC_50_ was achieved at a dose of 50 μg/ml, and after the concentration reached 150–450 μg/ml, the inhibitory effect of the extract on HSV-2 exceeded 3 logs (Chen et al., 2011). In addition to inhibiting NF-κB and restricting viral gene expression, the binding and penetration ability of the HSV-1, HSV-2, and acyclovir-resistant HSV-1 viruses at the initial stage of infection is also weakened by the extract, and the replication process of HSV is attenuated. Further research showed that the inhibitory activity of NF-κB involves contributions of the components quercetin and isoquercitrin, and quercetin suppressed the invasion ability of the virus (Hung et al., 2015). Moreover, a monkey kidney cell line (Vero cells) and swine testis cells (ST) were used to investigate the inhibitory activity of *H. cordata* in pseudorabies herpesvirus (PrV). In the Vero cell model, the infection rate was reduced by 70% after treatment with the *H. cordata* extract at a concentration of 2 mg/ml, while infectivity of the virus was completely suppressed at a concentration of 250 mg/ml. In contrast, the same concentration of extract exerted lower infection inhibitory activity on ST cells than Vero cells; nevertheless, single use of high-dose *H. cordata* extract showed apoptosis-inducing activity (Ren et al., 2011).

Furthermore, the new flavonoids houttuynoids A-E and houttuynoids G-J were isolated from the whole *H. cordata* plant, and both groups have been shown to possess anti-herpes virus activity. Through Vero cell experiments, houttuynoid A and houttuynoids G-J inhibited HSV-1 infection. The IC_50_ values of houttuynoids G-J were 38.46, 14.10, 62.00, and 70.76 µM, while houttuynoid A showed a lower value at 33.5 μM in the β-galactosidase activity assay. Moreover, the activity of herpes simplex virus type 2 and varicella-zoster virus was suppressed by houttuynoid A. Plaque reduction experiments and luciferase activity assays proved this effect (Chen et al., 2012; Chen S. D. et al., 2013; Li T. et al., 2017). Moreover, houttuynoid M demonstrated similar activity to houttuynoid A, and plaque formation experiments revealed that the IC_50_ value of houttuynoid M at 17.72 μM could suppress the activity of HSV-1 (Li JJ. et al., 2017).

#### Anti-Influenza Virus Activity

Human influenza viruses are divided into three types, A, B, and C, and the influenza A virus is the pathogen that causes the body to catch a cold (Luo, 2012). In the determination of neuraminidase activity, *H. cordata* showed effective anti-influenza virus activity and completely inhibited viral neuraminidase at a concentration of 250 mg/ml (HAN et al., 2016). Moreover, *in vivo* and *in vitro* experiments revealed the activity of *H. cordata* flavonoids against influenza virus H1N1, the survival rate and life span of mice infected with H1N1 were significantly improved through the combined action of rutin, hyperoside, isoquercitrin, and quercitrin in the extract, 50–200 μg/ml extract effectively reduced the H1N1 virus titre in the lung tissue, and neuraminidase activity was inhibited both in *in vivo* and *in vitro* experiments (Ling et al., 2020). Moreover, quercetin-3-rhamnoside (Q3R) obtained from *H. cordata* attenuated the replication of influenza A/WS/33 virus, which is associated with the indirect effect of Q3R on virus particles. Through the cytopathic effect, it was observed that Q3R significantly reduced the production of cytopathic changes. Compared with oseltamivir, Q3R showed stronger anti-A/WS/33 virus activity (Choi et al., 2009). The above findings suggested that flavonoids might be the main components acting against different influenza viruses.

#### Anti-Coronavirus Activity

Coronavirus is a type of virus widely distributed in nature, and it selectively infects humans and other vertebrates. At present, seven kinds of coronaviruses are known to be infectious to humans. Among them, the newly discovered severe acute respiratory syndrome coronavirus 2 (SARS-CoV-2) is circulating worldwide and has caused millions of deaths (Adhikari et al., 2020). Main protease (Mpro), papain-like protease (PLpro) and ADP ribose phosphatase (ADRP) are the three main replication proteins of SARS-CoV-2, and molecular docking by using Epic, LigPrep and Glide module of Schrödinger suite 2020–3 have shown that the metabolite (ligand) 6-hydroxyondansetron possesses binding affinity towards the receptors Mpro (PDB ID 6LU7) and PLpro (PDB ID 7JRN) with the best Glide scores (G-score) of −7.274 and −5.672, while quercitrin also showed binding affinity towards ADRP (PDB ID 6 W02) with a G-score −6.788. Furthermore, these compounds showed potential inhibition of Mpro and PLpro of SARS-CoV-2 without causing toxicity, although quercitrin showed fewer drug-like properties but demonstrated potential as an inhibitor for ADRP, and the results indicated the potential therapeutic activity of *H. cordata* (Das et al., 2021); however, the reports seems idealized, and more research is needed.

In research against severe acute respiratory syndrome coronavirus (SARS-CoV), enzymes and immune regulation play important roles. Aqueous extracts of *H. cordata* at 0–400 μg/ml effectively promoted the proliferation of mouse spleen lymphocytes in a dose-dependent manner, and increased expression of IL-2 and IL-10 in splenic lymphocytes was observed. The ratio of CD4^+^ and CD8^+^ T cells was also upregulated to enhance the body’s immunity. Moreover, the activity of 3C-like protease and RNA polymerase is critical for virus replication and was significantly inhibited in a dose-dependent manner at 0–1,000 μg/ml (Lau et al., 2008). Researchers used mouse hepatitis virus (MHV) as a coronavirus model to determine the anti-infective activity of the ethyl acetate extract of *H. cordata*, and treatment with the extract solution significantly inhibited the activity of MHV at the stage of virus infection (IC_50_ = 0.98 mg/ml), while a high dose of 2000 mg/kg did not show acute cytotoxicity (Chiow et al., 2016); however, human tolerance has not been effectively investigated.

**FIGURE 5:**
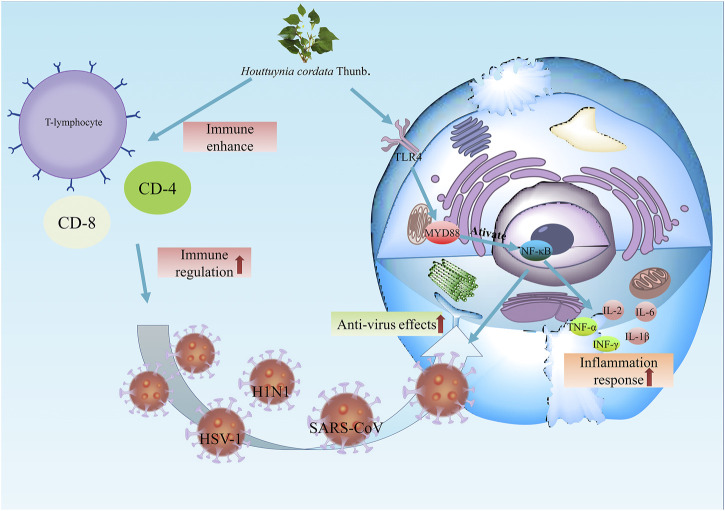
The antiviral effects of *H. cordata*. On the one hand, *H. cordata* enhances immune activity by activating T cells, CD4^+^ cells and CD8^+^. On the other hand, *H. cordata* combats viruses such as H1N1, HSV-1 and SARS-CoV by upregulating TLR4/NF-κB signalling.

Moreover, *H. cordata* exerts inhibitory activity against the avian infectious bronchitis virus, similar to coronavirus. Through the detection of plaque reduction and reverse transcription-polymerase chain reaction, 90% of viral infections were inhibited in Vero cells and chicken embryo kidney cells, and more than half of viral invasion was inhibited (Yin et al., 2011) (Figure 5).

### Antibacterial Activity

#### Anti-*Staphylococcus aureus* Activity

*Staphylococcus aureus*, a classical Gram-positive bacterium, usually parasitizes human and animal skin, the nasal cavity, gastrointestinal tract and other parts and is a common food-borne pathogenic microorganism (Lowy, 1998). Sodium houttuyfonate and EDTA-Na_2_ synergistically showed inhibitory activity against MRSA. Mice that were not treated with sodium houttuyfonate in combination with EDTA-Na_2_ all died 12 days after being infected with MRSA. In contrast, the survival rate of mice in the experimental group treated with sodium houttuyfonate combined with EDTA-Na_2_ was 75% after 28 days of MRSA infection, which was much higher than the 50 and 43.75% survival rates of mice treated with sodium houttuyfonate and EDTA-Na_2,_ respectively (Huang et al., 2015). Moreover, the modified sodium houttuyfonate combined with other antibacterial drugs showed excellent antibacterial effects. In studying the synergistic use of sodium houttuyfonate with oxacillin, cephalosporin, meropenem, and netilmicin, the median fractional inhibitory concentrations monitored by the checkerboard method were all between 0.25 and 0.38. However, time-kill experiments showed that using half the MIC of sodium new houttuyfonate combined with oxacillin and netilmicin, which resulted in MICs that were lower than normal, could significantly decrease the number of viable colonies (Lu et al., 2013). Furthermore, with the long-term use of penicillin and methicillin to produce methicillin-resistant *Staphylococcus* aureus (MRSA), extraction of fresh *H. cordata* leaves with ethanol resulted in higher inhibitory activity than that of aqueous extracts and decoctions of *H. cordata*. By comparing the minimum inhibitory concentration (MIC) of extract on methicillin-sensitive *Staphylococcus aureus* (MSSA) and MRSA, the concentration range of both was 110 μg/ml∼1760 μg/ml, and MSSA and MRSA with MICs below 440 μg/ml accounted for 70%. The inhibition mechanism may be related to preventing the formation of bacterial biofilms (Sekita et al., 2016).

#### Anti-*Pseudomonas aeruginosa* Activity

*Pseudomonas aeruginosa* (*PA*) is a common Gram-negative bacterium that can easily cause infection in injured parts of the body and lead to aggravation of disease (Mielko et al., 2019). Sodium houttuyfonate has been shown to have anti-*PA* activity through reverse transcription-quantitative polymerase chain reaction. It was detected that the biosynthesis of alginate, an important substance for *PA* biofilm formation, was inhibited, which is related to the downregulation of the expression of synthesis-related *algD* and *algR* genes by sodium houttuyfonate. At the same time, after treatment, scanning electron microscopy observations revealed that the morphology of the bacteria changed, and the content of alginate in the bacterial biofilm was also reduced (Wu et al., 2015). Moreover, sodium houttuyfonate and EDTA-Na_2_ synergistically enhanced the anti-*PA* activity. After mice were treated with sodium houttuyfonate and EDTA-Na_2_ separately for 28 consecutive days, the mortality rate of the mice was 75 and 81.25%, but after 28 days of combined treatment, the case fatality rate was only 43.75% (Huang et al., 2015). Interestingly, Wu et al. proved that sodium houttuyfonate inhibited PA activity through quorum sensing (QS), which is a method of information exchange between bacteria, using transmitted signal molecules to control the population size. *N-*Acyl homoserine lactone (AHL) is a signalling molecule in the *PA* population. In a previous study, sodium houttuyfonate effectively inhibited the synthetic lasl gene of AHL and reduced the level of expression, and the AHL receptor and the transcriptional regulator LasR were also inhibited, thereby downregulating the expression of the virulence factors pyocyanin and LasA; therefore, the *PA* population size can be effectively controlled through AHL mediation (Wu et al., 2014). Moreover, in a treatment evaluation of sodium houttuyfonate, the expression of the *rhll* and *pqsA* genes, which play key roles in the QS system, was significantly reduced and interfered with the production of pyocyanin; additionally, biofilm formation was monitored, and with the exception of *lasA*, the expression of the *LasB*, *LecA*, *phzM, pqsA,* and *pilG* genes was affected, which further inhibited the activation of *PA* virulence factors and biofilm formation (Zhao et al., 2020). These research results provide new insights for anti-*PA* activity.

## Toxicity

As an edible plant, the potential toxicity of *H. cordata* is usually ignored; however, recently, some studies reported that liver cancer is associated with aristolochic acid and aristolactams (Ng et al., 2017), and investigation of the toxicity mechanism of aristolochic acid in microphysiological systems showed that the specific metabolism of aristolochic acid in hepatocytes increased the cytotoxicity of the proximal tubule epithelial cells of the kidney (Chang et al., 2017); which caused people to be concerned about the safety of this plant due to it contains some aristolactam components (Ahn et al., 2017). However, aristolochic acid II is highly toxic *in vivo* due to its mutagenicity but is slightly toxic in *in vitro* cell models and toxicological studies in cell experiments can hardly reflect the true metabolism in the body (Michl et al., 2014). Furthermore, experiments *in vivo* with 95% ethanol extract of *H. cordata* directly demonstrated its potential toxicity, and following oral administration of a single dosage of 2000 mg/kg, no pathological reaction was observed in the rats during 14 days; however, during 28 consecutive days of oral administration of 500–1,000 mg/kg/d, a small number of rats died after 15 days, and histopathological analysis of organ slices showed that vacuum degeneration and inflammatory cell infiltration in liver tissue was present in the 1,000 mg/kg group, compared with the oral administration of ionized water, and a high dose in the kidney caused focal necrosis of renal epithelial cells, although no pathological signs in other organs were observed (Chen H. et al., 2021).

Although the above studies have shown the weak potential toxicity of *H. cordata*, there are no reports about its toxicity in a long-term consumption as vegetable and alone medicinal use in some areas of China and India, and it was listed as one of the plants that can be used as both food and medicine by the National Health Commission of China in 2013, indicating that *H. cordata* is relatively safe for oral administration in humans. Therefore, more sufficient and reliable data is needed to reveal its potential toxicity.

## Discussion and Conclusion

*H. cordata* is a medicinal plant with diverse biological activities. In the studies of organ protective activity, antioxidant stress and inflammation are important properties determining its therapeutic potential (Shingnaisui et al., 2018), inflammation and oxidative stress of the lung, heart, kidney and liver are alleviated. However, aristolochia derivatives in *H. cordata* seem to show nephrotoxicity, which is not consistent with its protective activity, different dosages may be one reason. It is also suggesting that the research on the nephrotoxic components and mechanisms of *H. cordata* is insufficient. Similarly, in the studies of alleviating liver injury and anti-liver tumour activity, the active components of *H. cordata* are not clear, and the research on the metabolism of active components seems to be beneficial for *H. cordata* to exert its hepatoprotective activity in diet. Overall, according to the reported literature, *H. cordata* appears to demonstrate selectivity for lung tissue, alleviating the processes of pneumonia (Lee et al., 2015), lung injury (Shi et al., 2020), pulmonary fibrosis (Ng et al., 2007) and lung tumour (Lou et al., 2019), which might be supported by the theory of traditional Chinese medicine. In comparison, *H. cordata* shows a shortage in the treatment of digestive and cardiovascular diseases. In studies of LPS- and virus-induced inflammation *in vivo* and *in vitro*, water extract and ethanol extract of *H. cordata* and separated flavonoids, volatile oil, sodium houttuyfonate and polysaccharide components all showed effective inhibition of the release of inflammatory factors, which might play an important role in alleviating inflammation, acute lung injury, heart remodelling and other pathological changes in tissue. Moreover, the NF-κB and TGB-β1/Smad signalling pathways are involved in, and whether *H. cordata* regulates the inflammatory response through other signalling pathways, only a few studies have been performed. In antitumour research, inducing apoptosis and cell cycle arrest are important characteristics of *H. cordata* against lung tumour, liver tumour, gastric carcinoma, colon tumour, and breast tumour. However, we found that components of the extracts used in the studies are not clear, and it also have not been fully characterized, which is a common lack in the antitumour activity and even the whole pharmacological studies of *H. cordata*. The experiments of pharmacological research should be carefully designed, strictly carried out, detailed records, and appropriate models and accurate determination methods are necessary (Heinrich et al., 2020). Therefore, we suggest that the pharmacological experiments *in vivo* and *in vitro* of *H. cordata* should follow the qualitative standards, and more research in disease prevention and treatment in diet is needed.

*H. cordata* also shows potential in combination with other drugs. As a traditional basic antiviral and antibacterial Chinese medicine, research on *H. cordata* eye drops combined with olopatadine hydrochloride in the treatment of vernal keratoconjunctivitis revealed the synergistic effect of this combination (Xu and Cai, 2019). Additionally, a clinical trial involving *mangosteen*, *Lithospermum officinale*, *Tribulus terrestris* L., and *H. cordata* extracts in the treatment of mild to moderate acne showed that inflammation and noninflammatory skin lesion counts were significantly reduced (Yang et al., 2021). More importantly, the synergistic use of *H. cordata* shows effective activity in alleviating diabetes insulin resistance (Wang et al., 2017) and anticancer pain (Wan et al., 2016), anti-bacterial and auxiliary cell transplantation, which may be an important aspect of the research on the therapeutic activity of *H. cordata* in the future. It is noteworthy that the adverse event evaluation of *H. cordata* injection showed that anaphylactic shock caused in the treatment, and synergistic use of penicillins, cephalosporins and macrolides increases the risk of allergic reactions (Wang et al., 2010). Due to the various phytochemical components of *H. cordata*, its pharmacological activity seems too optimistic, and its potential risks should be carefully studied. A preparation technology that used macroporous resin to extract the essential oil of *H. cordata* and then embedded the microemulsion to improve its biological activity and safety suggests the direction of the future development of *H. cordata* and its derivative compounds as agents (Pang et al., 2017), and the combination of *H. cordata* and a drug delivery system is expected to further enhance its potential in treatment.

Furthermore, *H. cordata* showed effective mitigating activity in various diseases, but it cannot be ignored of its liver toxicity and nephrotoxicity, which seem to appear at a high dose; therefore, research based on toxicology and pharmacology still needs to be strengthened to promote its role as an agent in the treatment of diseases. In addition, the pharmacological studies of the compound *in vitro* and even *in vivo* are not evidence that it can be converted into a drug to play a therapeutic role. Structural modification based on effective natural ingredients is expected to help reduce toxicity and enhance therapeutic activity, such as the β-elemene component in the rhizome of Wenyujin has been approved by the China Food and Drug Administration (CFDA) to treat a variety of cancers (Bai et al., 2021). Therefore, in research on the bioactive components of *H. cordata*, structural modification may be an aspect that needs to be paid attention to. Overall, studying the components and its pharmacological, toxicological activity of *H. cordata*, and to provide more data to eliminate potential risks; and also reasonable synergistic use and structural modification of compounds are still important topics for future research.
